# Improving quantum metrology protocols with programmable photonic circuits

**DOI:** 10.1515/nanoph-2024-0640

**Published:** 2025-02-20

**Authors:** Alberto Muñoz de las Heras, Diego Porras, Alejandro González-Tudela

**Affiliations:** 518690 Institute of Fundamental Physics IFF-CSIC, Calle Serrano 113b, 28006, Madrid, Spain

**Keywords:** photonic quantum metrology, variational quantum algorithms, programmable photonic circuits, non-linear optics and photonics

## Abstract

Photonic quantum metrology enables the measurement of physical parameters with precision surpassing classical limits by using quantum states of light. However, generating states providing a large metrological advantage is hard because standard probabilistic methods suffer from low generation rates. Deterministic protocols using non-linear interactions offer a path to overcome this problem, but they are currently limited by the errors introduced during the interaction time. Thus, finding strategies to minimize the interaction time of these non-linearities is still a relevant question. In this work, we introduce and compare different deterministic strategies based on continuous and programmable Jaynes–Cummings and Kerr-type interactions, aiming to maximize the metrological advantage while minimizing the interaction time. We find that programmable interactions provide a larger metrological advantage than continuous operations at the expense of slightly larger interaction times. We show that while for Jaynes–Cummings non-linearities the interaction time grows with the photon number, for Kerr-type ones it decreases, favoring the scalability to big photon numbers. Finally, we also optimize different measurement strategies for the deterministically generated states based on photon-counting and homodyne detection.

## 1 Introduction

Photonic quantum metrology [1], [2], [3], [4] uses quantum states of light to provide an advantage in the estimation of an unknown parameter *φ* beyond the one achievable with classical resources. The standard configuration for this purpose is the so-called Mach–Zehnder interferometer (MZI) in which two light probes are sent into a beam splitter, after which a phase difference *φ* is encoded between the two paths, which are afterwards mixed again in another beam splitter before measuring some observable. With classical light, the estimation error is always lower bounded by the standard quantum limit (SQL), i.e., $\Delta \varphi >1/\sqrt{N}$ , with *N* being the mean photon number of the state. On the contrary, using quantum states of light, like twin Fock states (TFS) |*N*/2, *N*/2⟩ [5], one can obtain estimation errors below the SQL, in this case $\Delta \varphi =\sqrt{2/N\left(N+2\right)}$ . The ultimate precision bound is Δ*φ* = 1/*N*, known as the Heisenberg limit (HL), which can be obtained if NOON states, $|NOON〉=\left(|N,0〉+|0,N〉\right)/\sqrt{2}$ , come out after the first beam splitter of the MZI. However, obtaining such a quantum metrological advantage in photonics is still an outstanding challenge. First, because generating metrologically useful quantum states with large photon numbers is hard. And second, because finding the optimal measurement scheme to attain such an advantage is a remarkable challenge as it depends on the probe states and it can be difficult to implement experimentally.

Most popular methods to generate metrologically useful states with large photon numbers are based on combining single photons through post-selection [6], [7], [8], [9], [10], [11], [12], [13], [14], [15], [16]. However, they suffer from decreasing efficiency rates with increasing photon numbers. Deterministic protocols based on non-linear interactions [17], [18], [19], [20], [21], [22], [23], [24], [25], [26], [27], [28], [29], [30] offer a path to avoid this problem, but suffer from low fidelities due to the errors accumulated during the interaction time. Recent proposals [31], [32] can lead to large fidelities even for large systems, but still decreasing with the photon number. Inspired by recent experimental [33], [34], [35] and theoretical [36], [37], [38], [39] research, we recently pointed out in Ref. [40] that optimizing programmable photonic circuits, i.e., combining quenches of photon tunnelings and non-linear interactions, can reduce the number of operations to generate metrologically relevant states with respect to other deterministic protocols [17], [18], [19], [20], [22], [23], [24], [25], [26], [27], [28], [29], [31], [32]. However, there were still several open questions:

– Does the reduced number of operations imply smaller interaction times? This is important as large interaction times lead to the accumulation of errors and thus to a decrease of fidelities.
– What is the nature of the generated states? Are they similar to known optimal states, such as NOON ones?
– Can the programmable circuits improve the performance of feasible measurements?

In this work, we answer these questions by benchmarking the programmable strategy of Ref. [40] in both the generation and measurement challenges of the photonic quantum metrology problem. We consider programmable photonic circuits with two classes of non-linearities: a Jaynes–Cummings (JC) interaction, like the one appearing in cavity QED [41], and a Kerr-type interaction [42]. However, differently from Ref. [40], we analyze in detail the more physically relevant interaction time, and also compare this programmable approach with the conceptually simpler continuous time evolution [22], [31], [32] under the non-linearity. This allows us to establish when the programmable approach is advantageous, but also to illustrate the origin of the unexplained scaling behavior found in Ref. [40]. In particular, we show that even though continuous operations can generate probe states featuring a quantum advantage in parameter estimation, programmability can lead to states approaching the HL while requiring similar interaction times. Finally, we also study the effect of optimizing the programmable photonic circuit before two types of measurements: photon counting [43], [44] and homodyne photon detection [45], [46], [47], finding an improvement in both cases, although less pronounced for the latter measurement strategy.

The manuscript is organized as follows: In Section 2, we define the physical setup of photonic quantum metrology, the programmable and continuous strategies that we employ, and the mathematical tools to quantify their metrological advantage. In Section 3, we study the generation of probe states using the continuous unitary time evolution under fixed photonic non-linearities. In Section 4, we benchmark these results with the preparation of probe states employing programmable photonic quantum circuits with tunable non-linearities. In Section 5, we analyze the optimization of the measurement part. Finally, our findings are summarized in Section 6.

## 2 General concepts of photonic quantum metrology

**Figure 1:**
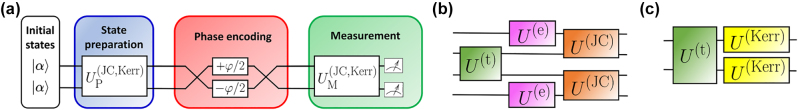
Overview of the continuous and programmable protocols. (a) Sketch of the estimation process involving two photonic modes. Two coherent initial states |*α*⟩ are employed as the input of a state preparation step that involves the unitary operator $U_{\text{P}}^{\left(JC,Kerr\right)}$ . In the continuous approach, this represents the time evolution under the Jaynes–Cummings (JC) or Kerr Hamiltonian. In the programmable approach, it corresponds to a parametrized quantum circuit (PQC) determined by an optimization loop aimed at maximizing the quantum Fisher information (QFI). The prepared state is then sent through a Mach–Zehnder interferometer (MZI) where a phase difference *φ* is encoded between the two modes. While in the continuous approach the measurement takes place immediatedly after phase encoding, in the programmable approach one introduces an additional PQC described by the unitary $U_{\text{M}}^{\left(JC,Kerr\right)}$ to prepare an optimal measurement. In this step, one aims at maximizing the classical Fisher information (CFI). (b, c) Sketches of the quantum circuits employed as $U_{\text{P}}^{\left(JC,Kerr\right)}$ and $U_{\text{M}}^{\left(JC,Kerr\right)}$ in the programmable approach: (b) represents the JC ansatz (where the two inner wires correspond to the photonic modes and the two outer wires are the emitters), while (c) corresponds to the Kerr ansatz.

Here, we start by reviewing in Section 2.1 the definition of the classical and quantum Fischer information, which we employ throughout this manuscript to characterize the metrological power of the quantum states of light we generate. Then, we describe the three parts of photonic quantum metrology setups (see Figure 1a): probe state preparation (in Section 2.2), parameter encoding (in Section 2.3), and measurement scheme (in Section 2.4), explaining in each of them the continuous and programmable non-linear time evolutions. Further information about the numerical simulations employed to calculate the results of our manuscript can be found in Sec. SM1 of the supplementary material (SM). Codes to reproduce the results of this manuscript are available in [48].

### 2.1 Definition of classical and quantum Fisher information

The ultimate precision limit of a certain probe state described by a density matrix *ρ* is quantified by the *quantum Fisher information* (QFI) [49], [50], which is independent of the measurement choice. If the interaction between probe and system is unitary, i.e., *ρ*_*φ*_ = *e* ^−*iHφ*^ *ρe* ^*iHφ*^ where *H* is a Hermitian operator, the QFI is independent of the unknown parameter *φ* and one can efficiently calculate it using the expression [4], [50], [51]

(1) $$F_{\text{Q}}=8\underset{\delta \to 0}{\lim}\frac{1-F\left[\rho_{\varphi },\rho_{\varphi +\delta }\right]}{\delta^{2}}\;$$

being $F\left(\rho_{1},\rho_{2}\right)=\mathrm{Tr}\left[\sqrt{\sqrt{\rho_{1}}\rho_{2}\sqrt{\rho_{1}}}\right]$ the fidelity between states *ρ*_1_ and *ρ*_2_.

For a particular measurement scheme, the attainable precision is given by the *classical Fisher informaction* (CFI) [52]

(2) $$F_{\text{C}}\left(\varphi \right)=\underset{\lambda }{\sum }P\left(\lambda |\varphi \right){\left(\frac{\partial \log\left[P\left(\lambda |\varphi \right)\right]}{\partial \varphi }\right)}^{2},$$

where *P* (*λ*|*φ*) is the probability to obtain a measurement outcome *λ* given a parameter value *φ*.

The minimum estimation error attainable by a certain state is given by ${\left(\Delta \varphi \right)}^{2}=1/F_{\text{Q}}$ . Similarly, an optimal measurement satisfies $F_{\text{Q}}=F_{\text{C}}$ . Such a hierarchy is summarized by the *quantum Cramer–Rao bound* [1], [4]

(3) $${\left(\Delta \varphi \right)}^{2}\geq \frac{1}{\nu F_{\text{C}}}\geq \frac{1}{\nu F_{\text{Q}}},$$

where *ν* is the number of independent repetitions of the estimation process.

In photonic quantum metrology, an archetypical problem consists on determining an unknown phase difference *φ* between two photonic modes forming the arms of an MZI, see Figure 1a. Let us now explain in detail the different steps of the protocol.

### 2.2 Probe preparation

For the preparation stage, we consider the application of unitary time evolutions given by two types of photonic non-linearities to easy-to-prepare states. The first type of non-linearity considered is provided by the interaction of each photonic mode with a single two-level emitter, like in the Jaynes–Cummings (JC) model of cavity QED setups [53], [54], while the other one is a photon–photon Kerr-type non-linearity [42]. They are described by the following Hamiltonians, respectively:

(4) $$H^{\left(JC\right)}=g\overset{2}{\underset{i=1}{\sum }}\left(\sigma_{i}^{†}a_{i}+\sigma_{i}a_{i}^{†}\right),$$

(5) $$H^{\left(Kerr\right)}=K\overset{2}{\underset{i=1}{\sum }}{\left(a_{i}^{†}a_{i}\right)}^{2},$$

where *g* and *K* are the interaction strength in each case, *σ*_*i*_ = |*g*⟩_*i*_⟨*e*|_*i*_ is a lowering operator for the emitter interacting with the *i*-th cavity, and $a_{i}^{\left(†\right)}$ are the annihilation (creation) operators for the photonic mode *i*.

For both non-linearity types, the two photonic modes are initialized in easy-to-prepare states, i.e., two coherent states |*α*⟩ ⊗|*α*⟩ = |*α*, *α*⟩ with the same mean photon number |*α*|^2^ = *N*/2, such that the total mean photon number is *N*. Besides, in the JC case, we consider the emitters initially in their ground states. Thus, the initial states for the two situations read $|\psi_{0}^{\left(JC\right)}〉=|g〉⊗|g〉⊗|\alpha ,\alpha 〉=|g,g,\alpha ,\alpha 〉$ and $|\psi_{0}^{\left(Kerr\right)}〉=|\alpha ,\alpha 〉$ , respectively.

We study two different ways of applying these non-linearities to the initial states:

– Letting the initial coherent states continuously evolve through the unitary dynamics of the non-linear Hamiltonians of Eqs. (4) and (5). The probe state is then given by $|\psi_{\text{P}}^{\left(JC,Kerr\right)}〉=e^{-iT^{\left(JC,Kerr\right)}H^{\left(JC,Kerr\right)}}|\psi_{0}^{\left(JC,Kerr\right)}〉$ , where *T* ^(JC,Kerr)^ is the physical evolution time for each non-linearity type. We define $\tilde{g}=T^{\left(JC\right)}g$ and $\tilde{K}=T^{\left(Kerr\right)}K$ as the adimensional parameters accounting for the interaction time. We label this strategy the *continuous* approach.
– Dividing the total evolution time in discrete time steps and applying the non-linearities in quenches, alternating them with linear photon tunneling Hamiltonians $H^{\left(t\right)}=J\left(a_{2}^{†}a_{1}+a_{1}^{†}a_{2}\right)$ , and, in the JC case, also with free evolution Hamiltonians $H^{\left(e\right)}=\Delta \sum_{i=1}^{2}\sigma_{i}^{†}\sigma_{i}$ to account for potential phase differences between the emitter and photonic modes. The resulting state after the quenched evolution can be written as $|\psi_{\text{P}}^{\left(JC,Kerr\right)}〉=U_{\text{P}}^{\left(JC,Kerr\right)}|\psi_{0}〉$ , where the unitary $U_{\text{P}}^{\left(JC,Kerr\right)}$ depends on the non-linearity applied. For the JC non-linearity, it reads:
(6) $$U_{\text{P}}^{\left(JC\right)}=\overset{d}{\underset{j=1}{\prod }}U_{j}^{\left(JC\right)}U_{j}^{\left(e\right)}U_{j}^{\left(t\right)},$$
  where *d* is the number of layers or quenches, $U_{j}^{\left(t\right)}=e^{-iT_{j}^{\left(t\right)}H^{\left(t\right)}}$ , $U_{j}^{\left(e\right)}=e^{-iT_{j}^{\left(e\right)}H^{\left(e\right)}}$ , and $U_{j}^{\left(JC\right)}=e^{-iT_{j}^{\left(JC\right)}H^{\left(JC\right)}}$ . For the Kerr-non linearity we build the unitary as:
(7) $$U_{\text{P}}^{\left(Kerr\right)}=\overset{d}{\underset{j=1}{\prod }}U_{j}^{\left(Kerr\right)}U_{j}^{\left(t\right)}.$$
  where $U_{j}^{\left(Kerr\right)}=e^{-iT_{j}^{\left(Kerr\right)}H^{\left(Kerr\right)}}$ . We remark that the layers are successively applied without any other kind of evolution taking place between them.
  The key idea of this *programmable* approach is that the unitaries $U_{\text{P}}^{\left(JC,Kerr\right)}\left(\pi \right)$ can be considered as *parametrized quantum circuits* (PQCs), i.e., *variational ansätze*. We refer to this step of the estimation process as the *preparation* PQC. The two types of ansätze (labeled JC and Kerr) considered in this manuscript are sketched in Figure 1b and c. The parameters of the PQCs ***π*** are optimized to minimize a given cost function. By choosing $C_{\text{P}}\left(\pi \right)=-F_{\text{Q}}\left(\pi \right)$ as the cost-function we find the optimal parameters ***π***_opt_ such that the resulting state after the unitary $|\psi_{\text{P}}^{\left(JC,Kerr\right)}〉=U_{\text{P}}^{\left(JC,Kerr\right)}\left(\pi_{\text{opt}}\right)|\psi_{0}〉$ has the maximum potential metrological advantage. In the case of the Kerr ansatz, we use $\left\{{\tilde{J}}_{j}=J_{j}T_{j}^{\left(t\right)}\right\}$ and $\left\{{\tilde{K}}_{j}=K_{j}T_{j}^{\left(Kerr\right)}\right\}$ as variational parameters. For the JC ansatz we employ $\left\{{\tilde{J}}_{j}=J_{j}T_{j}^{\left(t\right)}\right\}$ , $\left\{{\tilde{\Delta }}_{j}=\Delta_{j}T_{j}^{\left(e\right)}\right\}$ , and $\left\{{\tilde{g}}_{j}=g_{j}T_{j}^{\left(JC\right)}\right\}$ as variational parameters. We remark that the optimization of the variational parameters is an exclusive feature of the programmable approach, and that such optimization does not take place when one employs the continuous strategy. While the need for tunable photonic non-linearities in the programmable approach can be challenging, in Sec. SM2A of the SM we show that they can be implemented in state-of-the-art platforms. Experimental realizations of the programmable approach can be hindered by errors due to imperfect gate control, which are not as important in continuous time-evolution. However, we show in Sec. SM3 of the SM that typical values of these errors in current experimental setups do not modify our conclusions.

### 2.3 Phase encoding

The phase encoding takes place after the preparation phase. Here, we use the standard strategy of photonic quantum metrology, i.e., a phase difference between the two arms of the MZI, see Figure 1a. This is described by the unitary *U* ^(MZ) (^ *φ*) = *U* ^(BS)^ *U* ^(PD)^ (*φ*)*U* ^(BS)^, where $U^{\left(BS\right)}={\text{e}}^{-\text{i}\frac{\pi }{4}\left(a_{2}^{†}a_{1}+a_{1}^{†}a_{2}\right)}$ is the unitary of a symmetric beam splitter and $U^{\left(PD\right)}\left(\varphi \right)={\text{e}}^{-\text{i}\frac{\varphi }{2}\left(a_{2}^{†}a_{2}-a_{1}^{†}a_{1}\right)}$ encodes the phase difference between the two photonic modes. The output state after the phase encoding step is $|\psi_{\text{E}}^{\left(JC,Kerr\right)}\left(\varphi \right)〉=U^{\left(MZ\right)}\left(\varphi \right)|\psi_{\text{P}}^{\left(JC,Kerr\right)}〉$ .

### 2.4 Measurement

Finally, to estimate the unknown parameter one needs to measure the state coming from the encoding phase, i.e., $|\psi_{\text{E}}^{\left(JC,Kerr\right)}\left(\varphi \right)〉$ . Here, we will consider different strategies for the continuous and programmable approaches:

– In the continuous approach, the output state of the encoding stage $|\psi_{\text{E}}^{\left(JC,Kerr\right)}\left(\varphi \right)〉$ is directly employed to assess the metrological power of a specific measurement. This is quantified by the CFI $F_{\text{C}}\left(\varphi \right)$ , that requires calculating the probability distributions appearing in Eq. (2) as $P\left(\lambda |\varphi \right)=|\left\langle \lambda |\psi_{\text{E}}^{\left(JC,Kerr\right)}\left(\varphi \right)\right\rangle {|}^{2}$ , which depend on the type of measurement employed.
– For the programmable approach, we consider that the state after phase encoding $|\psi_{\text{E}}^{\left(JC,Kerr\right)}\left(\varphi \right)〉$ undergoes a second PQC, which we label *pre-measurement* PQC to distinguish it from the preparation PQC. Each layer of the pre-measurement PQC is formed by the same gates as a layer of the preparation PQC, as sketched in Figure 1b and c. However, the pre-measurement PQC is described by a unitary $U_{\text{M}}^{\left(JC,Kerr\right)}\left(\mu \right)$ , whose parameters (***μ***) are optimized to maximize the CFI for a given measurement. In the programmable approach, the optimizer computes the CFI from the probabilities $P\left(\lambda |\varphi \right)=|\left\langle \lambda |\psi_{\text{M}}^{\left(JC,Kerr\right)}\left(\varphi \right)\right\rangle {|}^{2}$ calculated with the state after the pre-measurement PQC, labeled as $|\psi_{\text{M}}^{\left(JC,Kerr\right)}\left(\varphi \right)〉=U_{\text{M}}^{\left(JC,Kerr\right)}\left(\mu \right)|\psi_{\text{E}}^{\left(JC,Kerr\right)}\left(\varphi \right)〉$ .

In this paper we consider two measurement types: photon counting and homodyne detection. Furthermore, for the JC non-linearity we assume that we also have access to the state of the quantum emitters coupled to the photonic modes. For the two approaches, one needs to define the correct positive operator-valued measure (POVM) to calculate the probability distributions *P* (*λ*|*φ*) of the CFI (2). For photoncounting:

– The POVM of the Kerr ansatz uses only the eigenstates {|*N*_1_, *N*_2_⟩} of the number operators $n_{i}=a_{i}^{†}a_{i}$ of the two photonic modes. From this, one obtains the probabilities $P\left(N_{1},N_{2}|\varphi \right)=|\left\langle N_{1},N_{2}|\psi_{\text{E}}^{\left(Kerr\right)}\left(\varphi \right)\right\rangle {|}^{2}$ .
– For the JC ansatz, one needs to include as well in the POVM the eigenstates *z*_*i*_ of the *σ*_z,i_ Pauli operators of the emitters, such that the final probabilities read $P\left(z_{1},z_{2},N_{1},N_{2}|\varphi \right)=|\left\langle z_{1},z_{2},N_{1},N_{2}|\psi_{\text{E}}^{\left(JC\right)}\left(\varphi \right)\right\rangle {|}^{2}$ .

In the case of homodyne detection:

– For the Kerr ansatz, one has to calculate the probabilities $P\left(x_{1}^{\left(\theta \right)},x_{2}^{\left(\theta \right)}|\varphi \right)=|\left\langle x_{1}^{\left(\theta \right)},x_{2}^{\left(\theta \right)}|\psi_{\text{E}}^{\left(Kerr\right)}\left(\varphi \right)\right\rangle {|}^{2}$ of obtaining a measurement outcome $x_{1}^{\left(\theta \right)}$ , $x_{2}^{\left(\theta \right)}$ when one measures the generalized quadrature $X_{i}\left(\theta \right)=\left({\text{e}}^{-\text{i}\theta }a_{i}^{†}+{\text{e}}^{\text{i}\theta }a_{i}\right)/\sqrt{2}$ of each mode *i* = 1, 2, where *θ* is the quadrature angle.
– For the JC ansatz, the probabilities are instead $P\left(z_{1},z_{2},x_{1}^{\left(\theta \right)},x_{2}^{\left(\theta \right)}|\varphi \right)=|\left\langle z_{1},z_{2},x_{1}^{\left(\theta \right)},x_{2}^{\left(\theta \right)}|\psi_{\text{E}}^{\left(JC\right)}\left(\varphi \right)\right\rangle {|}^{2}$ , since the POVM in this case is formed by the eigenstates of the *σ*_z,1_ ⊗ *σ*_z,2_ ⊗ *X*_1_(*θ*) ⊗ *X*_2_(*θ*) operator, i.e., $\left\{|z_{1},z_{2},x_{1}^{\left(\theta \right)},x_{2}^{\left(\theta \right)}〉\right\}$ .

Finally, let us note an important difference between the continuous and programmable approaches. In the former, we choose the optimal *θ* for which the CFI is maximized [see Sec. SM4 of the SM]. On the contrary, in the latter we fix *θ* = 0 since the pre-measurement PQC already allows to optimize the CFI [see Sec. SM5 of the SM]. In Sec. SM6 of the SM we also consider an alternative strategy based on fixing $U_{\text{M}}^{\left(JC,Kerr\right)}=1$ and optimizing the quadrature angle, finding similar results to those presented in the manuscript.

## 3 Probe state generation in the continuous approach

In this Section we study the probe states generated with the continuous evolution, focusing on the maximal potential metrological advantage that they can provide (given by the QFI). Section 3.1 analyzes the results for the JC non-linearity, while Section 3.2 focuses on the Kerr interaction. The results of this Section will serve as a baseline to benchmark the programmable approach in Section. 4.

### 3.1 JC non-linearity

**Figure 2:**
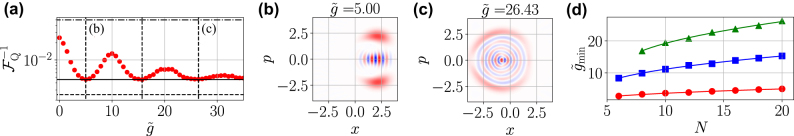
Continuous probe state preparation with the JC non-linearity. (a) Inverse QFI $F_{\text{Q}}^{-1}$ (red dots) for continously generated states using the JC non-linearity and an initial state with mean-photon number *N* = 20. Horizontal lines: dashed, solid, and dashed-dotted lines correspond to the HL, the TFS results, and the SQL, respectively. The vertical lines signal the position of the minima. (b, c) Wigner functions of the displaced cat state and the displaced Fock state that appear at the values of $\tilde{g}$ signalized by the first and third vertical lines in panel (a). (d) Interaction time ${\tilde{g}}_{\min}$ at which $F_{\text{Q}}^{-1}$ reaches its first (red circles), second (blue squares), and third (green triangles) minimum as a function of *N*. Circles are numerical results, while solid lines correspond to square-root fits.

We start with the states that can be generated using the JC non-linear Hamiltonian (4). Here, the input state $|\psi_{0}^{\left(JC\right)}〉=|g,g,\alpha ,\alpha 〉$ undergoes a unitary evolution given by $|\psi_{\text{P}}^{\left(JC\right)}\left(\tilde{g}\right)〉=e^{-iT^{\left(JC\right)}H^{\left(JC\right)}\left(g\right)}|\psi_{0}^{\left(JC\right)}〉$ , where $\tilde{g}=T^{\left(JC\right)}g$ is the adimensional interaction time. To assess the metrological power of $|\psi_{\text{P}}^{\left(JC\right)}\left(\tilde{g}\right)〉$ , we calculated its QFI for phase estimation in an MZI. In Figure 2a we show the inverse of the QFI, $F_{\text{Q}}^{-1}$ , for two initial coherent states featuring a total mean number of photons *N* = 20. The QFI displays an oscillatory behavior: $F_{\text{Q}}^{-1}$ first decreases down to the estimation error set by TFS with 10 photons in each mode, i.e., |10⟩ ⊗|10⟩. Then, $F_{\text{Q}}^{-1}$ increases again, but it does not recover its initial value due to the dephasing of the different photon-number components of the coherent states introduced by the non-linearity. Similar dumped oscillations are known to arise in the probability of measuring a single quantum emitter in its ground or excited state [53].

We now study the nature of the states produced by the protocol, particularly the ones minimizing $F_{\text{Q}}^{-1}$ , which provide the largest metrological power within the continuous approach with JC non-linearities. To do it, in Figure 2b and c we plot the Wigner quasiprobability distribution of a single photonic mode (obtained by tracing the other photonic mode as well as the two quantum emitters, i.e., ${\mathrm{Tr}}_{\mathrm{ee1}}\left\{\left|\psi_{\text{P}}^{\left(JC\right)}\left(\tilde{g}\right)〉〈\psi_{\text{P}}^{\left(JC\right)}\left(\tilde{g}\right)\right|\right\}$ ) at values of the adimensional interaction time $\tilde{g}≃5$ and $\tilde{g}≃26$ , corresponding to the first and third minima of $F_{\text{Q}}^{-1}$ . Note that, in general, the states generated at each minimum of $F_{\text{Q}}^{-1}$ are different. In particular, the Wigner distribution at such values of $\tilde{g}$ corresponds, respectively, to a displaced cat state along the *x* quadrature, and a displaced Fock state, also along the *x* quadrature as already shown in a previous work [31]. The second minimum of $F_{\text{Q}}^{-1}$ , appearing at $\tilde{g}≃16$ , corresponds to a state displaying phase-space oscillations but that cannot be associated with any well-known type. Despite their different nature, the states minimizing $F_{\text{Q}}^{-1}$ feature very similar values of the QFI.

As a next step, we calculated the dependence with *N* of the values of $\tilde{g}$ at which the minima of $F_{\text{Q}}^{-1}$ take place, which we label ${\tilde{g}}_{\min}$ . Figure 2d shows that, for the first three minima, ${\tilde{g}}_{\min}$ follows a $\sqrt{N}$ dependence. This is illustrated with a square root fit of the type ${\tilde{g}}_{\min}=\alpha \sqrt{N+\beta }+\gamma$ for each minimum, which reproduces the behavior of the data points with great precision. Actually, such a scaling with *N* was already observed for the displaced Fock state in Ref. [31]. However, an important observation of our analysis is that metrologically useful of states with the same potential that displaced Fock states can be obtained at shorter interaction times (red line versus green line in Figure 2d.

### 3.2 Kerr non-linearities

**Figure 3:**
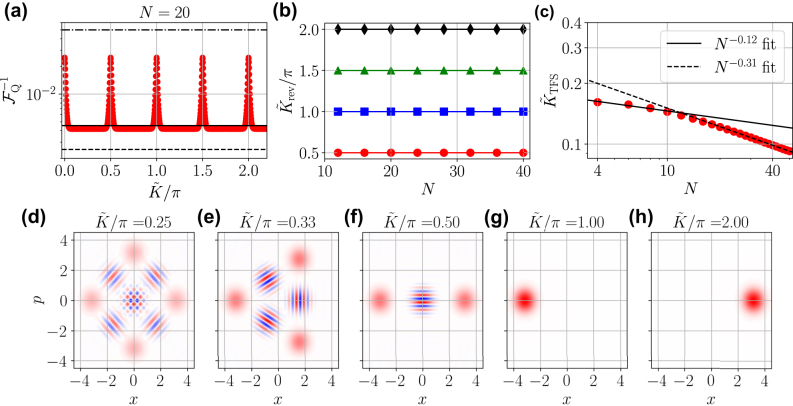
Continuous probe state preparation with the Kerr non-linearity. (a) Inverse QFI $F_{\text{Q}}^{-1}$ (red dots) for continuously generated states using the Kerr non-linearity and an initial state with mean-photon number *N* = 20. (b) Interaction time ${\tilde{K}}_{\text{rev}}$ of the first (red circles), second (blue squares), third (green triangles), and fourth (black rhombus) revivals of $F_{\text{Q}}^{-1}$ as a function of *N*. Solid lines are guides for the eye. (c) Interaction time ${\tilde{K}}_{\text{TFS}}$ necessary for the QFI of the generated probe states to equal that of TFS as a function of *N* (red circles). The lines correspond to linear fits. (d–h) Wigner function of a single photonic mode for *N* = 20 at several values of the interaction time $\tilde{K}$ .

We now repeat the previous analysis for an initial state $|\psi_{0}^{\left(Kerr\right)}〉$ evolving under the Kerr Hamiltonian *H* ^(Kerr)^ given by Eq. (5). The output state of the probe preparation stage reads

(8) $$|\psi_{\text{P}}^{\left(Kerr\right)}\left(\tilde{K}\right)〉=e^{-iT^{\left(Kerr\right)}H^{\left(Kerr\right)}\left(K\right)}|\psi_{0}^{\left(Kerr\right)}〉,$$

with $\tilde{K}=T^{\left(Kerr\right)}K$ being the adimensional interaction time. The output state $|\psi_{\text{P}}^{\left(Kerr\right)}\left(\tilde{K}\right)〉$ is fed to the MZI to compute the QFI. The results for $F_{\text{Q}}^{-1}$ as a function of the interaction time $\tilde{K}$ are shown in Figure 3a for a total mean photon number *N* = 20. In this case, the inverse of the QFI features a similar behavior to that observed for the JC interaction: starting from a large value corresponding to the initial coherent states, $F_{\text{Q}}^{-1}$ first decreases slightly below the error bound obtained by TFS |*N*/2⟩ ⊗|*N*/2⟩, and then increases again. However, differently from the JC interaction, the Kerr non-linearity allows the QFI to recover its initial value. Such a first revival of the QFI was already noticed by [32]. However, there are more revivals appearing at integer multiples of $\tilde{K}=\pi /2$ independently of *N*, as it is shown in Figure 3b, where we plot the first four revivals. The reason behind this behavior is that the *n* ^2^ non-linearity of the Kerr Hamiltonian allows for a rephasing of the photon-number components at such times resulting in either coherent states or 2-component cat states [see panels (f–h) for the Wigner function of a single mode at the first, second, and fourth revivals. At the third revival a 2-component cat state is generated]. However, none of these states provides the best potential metrological advantage.

The most promising states are actually generated in the plateaus between the QFI revivals of Figure 3a. Here the generated probe states $|\psi_{\text{P}}^{\left(Kerr\right)}\left(\tilde{K}\right)〉$ evolve between different multicomponent cat states. In particular, at values of the non-linearity time $\tilde{K}=\pi \left(\ell +1/q\right)$ , where *ℓ* = 0, 1, 2, … and *q* = … − 2, − 1, 1, 2, …, a *q*-component cat state is produced [22], [41]. For *q* ≥ 3 the *q*-component cat states feature a QFI comparable to that of TFS. This is shown explicitly in Figure 3d and e, where we plot the Wigner distribution for *ℓ* = 0 and *q* = 3, 4. For such values of $\tilde{K}$ , Figure 3a shows that these states feature an $F_{\text{Q}}^{-1}$ slightly smaller than that of TFS with the same *N*.

In analogy with what we do with the JC non-linearity, we study the interaction time ${\tilde{K}}_{\text{TFS}}$ that the Kerr non-linearity needs to transform the initial state $|\psi_{0}^{\left(Kerr\right)}〉=|\alpha ,\alpha 〉$ with |*α*|^2^ = *N*/2 into a state featuring the same value of the QFI as the TFS |*N*/2, *N*/2⟩. This is shown in Figure 3c. Differently from the JC model, ${\tilde{K}}_{\text{TFS}}$ decreases with *N*, meaning that larger probe states need smaller interaction times to attain the metrological advantage of TFS. In the case of the Kerr interaction, the dependence of ${\tilde{K}}_{\text{TFS}}$ with *N* is fit by two power laws: for *N* ≲ 10 we have *μ* = −0.12 and for *N* ≳ 10 we have *μ* = −0.31. Our intuition is that the quadratic dependence of the Kerr non-linearity on the photon number enables a faster non-trivial state preparation, although we cannot explain analytically the asymptotic behavior displayed in Figure 3c.

Summing up, for both the JC and the Kerr interaction the continuous application of the non-linearity allows one to obtain states with a QFI similar to TFS, but it does not allow to saturate the HL. In the next section, we show that the programmable approach offers a path to reach the HL using the same type of non-linearities and requiring comparable interaction times.

## 4 Probe state generation in the programmable approach

In this Section we apply the programmable approach described in Section 2.2 and compare it with the performance of the continuous approach. A detailed description of the optimization algorithm employed to determine the variational parameters of the photonic quantum circuit can be found in Sec. SM1 of the SM.

**Figure 4:**
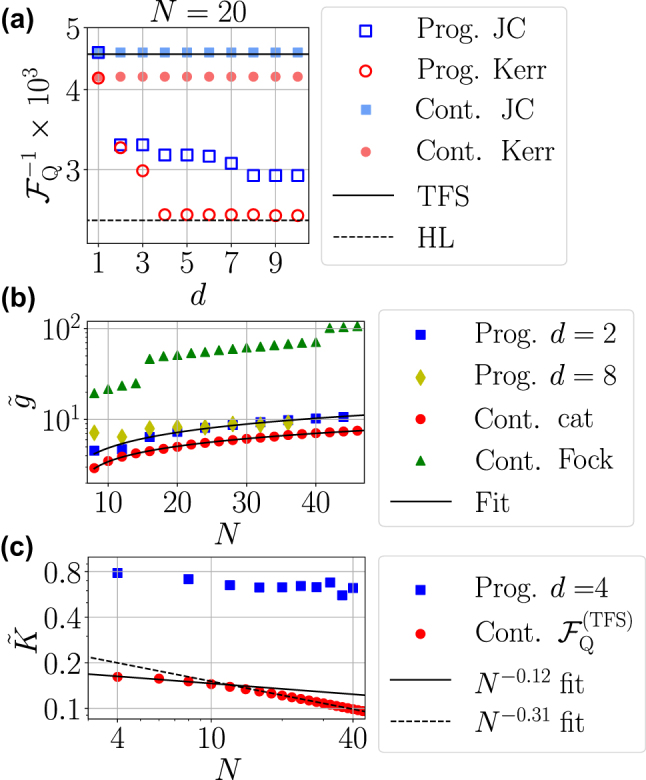
Programmable probe state preparation. (a) Inverse QFI $F_{\text{Q}}^{-1}$ as a function of the number of layers *d* in the JC (blue void squares) and Kerr (red void circles) ansätze of the programmable approach for a mean-photon number *N* = 20. The first minimum of $F_{\text{Q}}^{-1}$ obtained by the continuous approach employing a JC (Kerr) interaction is signaled by the solid blue squares (solid red squares). Although they do not depend on *d*, we plot them as points instead of lines for better figure clarity. Black dashed (solid) lines signal the HL (TFS results). (b) JC non-linearity: interaction time $\tilde{g}$ needed to produce states with the smallest value of $F_{\text{Q}}^{-1}$ as a function of *N* for each strategy. Blue squares (yellow rhombus): sum of the absolute value of all interaction optimal parameters ${\tilde{g}}_{\text{T}}$ obtained by the programmable approach with *d* = 2 (*d* = 8) Green triangles: displaced Fock states obtained with the continuous approach (data taken from Ref. [31]). Red circles: displaced cat states obtained with the continuous approach. Lines are square root fits. (c) Kerr non-linearity: interaction time $\tilde{K}$ needed to produce states with the smallest value of $F_{\text{Q}}^{-1}$ as a function of *N* for each strategy. Blue squares: sum of the absolute value of all interaction optimal parameters ${\tilde{K}}_{\text{T}}$ obtained by the programmable approach with *d* = 4. Red circles: interaction time ${\tilde{K}}_{\text{TFS}}$ necessary to produce states with the same QFI as TFS in the continuous approach. Lines are linear fits.

We start by benchmarking the values of QFI obtained with the two approaches to quantify the metrological advantage of the probe states generated in each case. In Figure 4a we show the inverse of the QFI, $F_{\text{Q}}^{-1}$ , for an initial state with a fixed mean-photon number *N* = 20 as a function of the number of layers *d* employed by the programmable approach. Results for the JC (Kerr) ansatz are shown as blue squares (red circles). For reference, we also plot in black solid and dashed lines the QFI obtained by TFS and the HL, respectively, and the best QFI obtained in the continuous approach for the JC (Kerr) non-linearity in filled (empty) triangles. The programmable approach is able to improve the results obtained by continuous evolution as we increase the number of layers of the circuit, even almost saturating the HL with only four layers in the case of the Kerr ansatz.

While this improvement with the number of layers (and thus with the number of operations) was already observed in our previous work [40], whether it comes at the price of larger total interaction times when considering all layers was still a relevant open question. The total interaction time can be calculated by summing the interaction times through all the circuit, i.e., ${\tilde{g}}_{\text{T}}=\sum_{i=1}^{d}|{\tilde{g}}_{i}|$ and ${\tilde{K}}_{\text{T}}=\sum_{i=1}^{d}|{\tilde{K}}_{i}|$ , for the JC and Kerr ansätze, respectively. It is important to have a total interaction time as small as possible because errors produced, e.g., by photon loss, depend on the total interaction time and not on the number of layers.

In Figure 4b and c, we address this question by showing the total interaction time required by the programmable and continuous approaches to reach their maximum QFI as a function of the mean photon number of the initial state. In Figure 4b we plot ${\tilde{g}}_{\text{T}}$ as a function of *N* for JC ansätze with *d* = 2 (*d* = 8) in blue squares (yellow rhombus). The values of ${\tilde{g}}_{\text{T}}$ for the same *N* are very similar irrespectively of the number of layers *d* of the ansatz, which means that the optimization can improve the QFI without increasing the total interaction time. A $\sqrt{N}$ -dependence similar to that observed in the continuous approach is found for ${\tilde{g}}_{\text{T}}$ irrespectively of the number of layers, although it becomes less clear as one increases *d*. To shine more light on the comparison between the continuous and the programmable approaches, we plot the time that the former strategy requires to generate displaced Fock (green triangles, data obtained by Ref. [31]) and cat states (red circles). The programmable approach requires total interaction times that lie between those of the two state classes generated with the continuous strategy. In Figure 4c we perform the same analysis for the Kerr non-linearity ansatz, and we benchmark the results of the optimal preparation PQC with *d* = 4 layers (in blue squares) against those obtained by continuous evolution (in red circles). Here we want to highlight two results: i) The programmable Kerr ansatz is able to saturate the HL at the expense of slightly larger interaction times than the continuous approach. ii) Like in the continuous strategy, the programmable interaction time also decreases with the photon number. This implies that Kerr-non linearities open a path to generate large photon states approaching the HL using the programmable approach. Remarkably, when the two ansätze are formed by a single layer, they produce output states similar to those generated in the continuous approach (namely, a displaced cat state for the JC non-linearity and a multi-component cat state for the Kerr one). However, for d ≥ 2, the output states found with the programmable strategy differ significantly from the continuously-generated ones.

Finally, note that the interaction times needed by the two strategies are within reach in state-of-the-art experimental platforms, as analyzed in Sec. SM2B of the SM.

## 5 Measurements

Let us finally focus on the last part of photonic quantum metrology protocols, that is, the measurement of the probe state to estimate the encoded parameter. In particular, we calculate the CFI for two different measurements, that are, photon counting and homodyne detection, as explained in Section 2.4.

**Figure 5:**
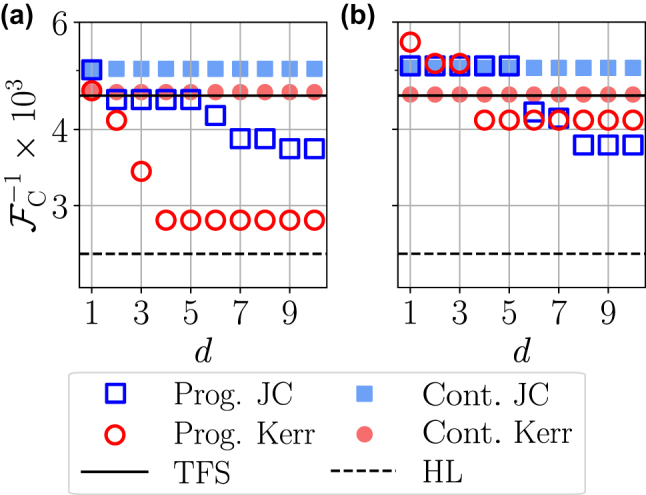
Continuous and programmable approaches for the measurement step. (a, b) Inverse CFI $F_{\text{C}}^{-1}$ as a function of the number of layers *d* in the JC (blue void squares) and Kerr (red void circles) ansätze of the programmable approach for a mean-photon number *N* = 20 for photon counting (a) and homodyne detection (b). The smallest values of $F_{\text{C}}^{-1}$ obtained by the continuous approach employing a JC (Kerr) interaction are signaled by the solid blue squares (solid red circles). Although they do not depend on *d*, we plot them as points instead of lines for better figure clarity. Black dashed (solid) lines signal the HL (TFS results).

In Figure 5a we show the CFI for photon-counting measurements corresponding to the best probe states generated by continuous JC and Kerr non-linear evolution (i.e., those that correspond to the first minima of $F_{\text{Q}}^{-1}$ in Figures 2a and 3a) in filled and empty triangles, respectively, for an initial state with mean-photon number *N* = 20. The Kerr evolution is able to obtain the CFI of the TFS, whereas the JC non-linearity performs slightly worse. However, the important part of this figure is the evolution of the CFI as a function of the number of layers *d* for both the JC (blue squares) and Kerr (red circles) programmable ansätze. Like for the QFI, both ansätze are able to go below the precision attainable by TFS, although in this case they do not saturate the HL.

In Figure 5b we carry a similar analysis for both the continuous and programmable approaches, but in this case by considering homodyne detection as the measurement protocol. The quadrature angle is chosen as explained in Section 2.4. For the continuously generated states, both the JC and the Kerr non-linearities give values of $F_{\text{C}}^{-1}$ very similar to those obtained using photon counting, approaching the CFI of TFS in the former case and saturating it in the latter. In the programmable approach, the pre-measurement optimization allows to reach larger values of the CFI than those of TFS. However, for this strategy homodyne detection performs worse than photon counting.

Note that, while photon counting outperforms homodyne detection at providing larger values of the CFI, conducting full photon counting tomography in a real experiment is a challenging task. In fact, to date, high photon number detection efficiency has only been demonstrated on the infrared using multiplexed single-photon detectors [43], [44]. Homodyne detection, on the other hand, is an experimentaly friendlier measurement technique.

## 6 Conclusions and outlook

Summing up, we present a systematic study of the potential of programmable photonic non-linearities to improve the generation of metrologically-useful quantum states of light. By studying the quantum Fisher information of the generated states, we demonstrate that the programmable strategy reaches better precisions than continuous time evolution using similar interaction times. When one employs the former strategy, while for the Jaynes–Cummings non-linearity the total interaction time grows with the number of photons, for the Kerr non-linearity the total interaction time decreases with increasing photon number, and the generated states approach the Heisenberg limit. Finally, we study the role of the measurement in the estimation process, finding that photon counting performs better than homodyne detection for the generated states, and we benchmark the improvement that adding a pre-measurement programmable quantum circuit produces. As an outlook, in future works we will explore other approaches for quantum state generation, including driven-dissipative settings [55], [56], [57], [58], [59] or by using other non-linearity types [60], [61], [62], [63], [64]. Another possible direction is to harness the programmable photonic circuits in the context of state preparation for bosonic error-correcting codes [65], [66], such as GKP states [67], [68], [69], [70], [71], which can also be useful for quantum metrology [72].

## Supplementary Material

Supplementary Material Details

## References

[1] Demkowicz-Dobrzański R., Jarzyna M., Kołodyński J., Wolf E. (2015). Chapter four - quantum limits in optical interferometry. *ser. Progress in Optics*.

[2] Dowling J. P., Seshadreesan K. P. (2015). Quantum optical technologies for metrology, sensing, and imaging. *J. Lightwave Technol.*.

[3] Pirandola S., Bardhan B. R., Gehring T., Weedbrook C., Lloyd S. (2018). Advances in photonic quantum sensing. *Nat. Photonics*.

[4] Polino E., Valeri M., Spagnolo N., Sciarrino F. (2020). Photonic quantum metrology. *AVS Quantum Sci.*.

[5] Holland M. J., Burnett K. (1993). Interferometric detection of optical phase shifts at the heisenberg limit. *Phys. Rev. Lett.*.

[6] Mitchell M. W., Lundeen J. S., Steinberg A. M. (2004). Super-resolving phase measurements with a multiphoton entangled state. *Nature*.

[7] Xiang G. Y., Higgins B. L., Berry D. W., Wiseman H. M., Pryde G. J. (2011). Entanglement-enhanced measurement of a completely unknown optical phase. *Nat. Photonics*.

[8] Israel Y., Afek I., Rosen S., Ambar O., Silberberg Y. (2012). Experimental tomography of noon states with large photon numbers. *Phys. Rev. A*.

[9] Yao X.-C. (2012). Observation of eight-photon entanglement. *Nat. Photonics*.

[10] Guerlin C. (2007). Progressive field-state collapse and quantum non-demolition photon counting. *Nature*.

[11] Deléglise S. (2008). Reconstruction of non-classical cavity field states with snapshots of their decoherence. *Nature*.

[12] Sayrin C. (2011). Real-time quantum feedback prepares and stabilizes photon number states. *Nature*.

[13] Wang X. L. (2016). Experimental ten-photon entanglement. *Phys. Rev. Lett.*.

[14] González-Tudela A., Paulisch V., Kimble H. J., Cirac J. I. (2017). Efficient multiphoton generation in waveguide quantum electrodynamics. *Phys. Rev. Lett.*.

[15] Zhong H.-S. (2018). 12-photon entanglement and scalable scattershot boson sampling with optimal entangled-photon pairs from parametric down-conversion. *Phys. Rev. Lett.*.

[16] Deng X. (2024). Quantum-enhanced metrology with large fock states. *Nat. Phys.*.

[17] Vogel K., Akulin V. M., Schleich W. P. (1993). Quantum state engineering of the radiation field. *Phys. Rev. Lett.*.

[18] Law C. K., Eberly J. H. (1996). Arbitrary control of a quantum electromagnetic field. *Phys. Rev. Lett.*.

[19] Hofheinz M. (2008). Generation of fock states in a superconducting quantum circuit. *Nature*.

[20] Hofheinz M. (2009). Synthesizing arbitrary quantum states in a superconducting resonator. *Nature*.

[21] Afek I., Ambar O., Silberberg Y. (2010). High-noon states by mixing quantum and classical light. *Science*.

[22] Kirchmair G. (2013). Observation of quantum state collapse and revival due to the single-photon kerr effect. *Nature*.

[23] González-Tudela A., Paulisch V., Chang D. E., Kimble H. J., Cirac J. I. (2015). Deterministic generation of arbitrary photonic states assisted by dissipation. *Phys. Rev. Lett.*.

[24] Zhang J., Um M., Lv D., Zhang J.-N., Duan L.-M., Kim K. (2018). Noon states of nine quantized vibrations in two radial modes of a trapped ion. *Phys. Rev. Lett.*.

[25] Li J.-P. (2020). Multiphoton graph states from a solid-state single-photon source. *ACS Photonics*.

[26] Walschaers M. (2021). Non-Gaussian quantum states and where to find them. *PRX Quantum*.

[27] Yang C.-W., Yu Y., Li J., Jing B., Bao X.-H., Pan J.-W. (2022). Sequential generation of multiphoton entanglement with a rydberg superatom. *Nat. Photonics*.

[28] Eickbusch A. (2022). Fast universal control of an oscillator with weak dispersive coupling to a qubit. *Nat. Phys.*.

[29] Long D. M., Crowley P. J. D., Kollár A. J., Chandran A. (2022). Boosting the quantum state of a cavity with floquet driving. *Phys. Rev. Lett.*.

[30] Chen Y., Elben A., Rubio A., Refael G. (2024). Bosonic entanglement and quantum sensing from energy transfer in two-tone floquet systems. ..

[31] Uria M., Solano P., Hermann-Avigliano C. (2020). Deterministic generation of large fock states. *Phys. Rev. Lett.*.

[32] Uria M., Maldonado-Trapp A., Hermann-Avigliano C., Solano P. (2023). Emergence of non-Gaussian coherent states through nonlinear interactions. *Phys. Rev. Res.*.

[33] He X. L. (2023). Fast generation of Schrödinger cat states using a kerr-tunable superconducting resonator. *Nat. Commun.*.

[34] Cimini V. (2024). Variational quantum algorithm for experimental photonic multiparameter estimation. *NPJ Quantum Inf.*.

[35] Nielsen K. H. (2024). Programmable nonlinear quantum photonic circuits. ..

[36] Krisnanda T., Ghosh S., Paterek T., Liew T. C. (2021). Creating and concentrating quantum resource states in noisy environments using a quantum neural network. *Neural Networks*.

[37] Steinbrecher G. R., Olson J. P., Englund D., Carolan J. (2019). Quantum optical neural networks. *npj Quantum Inf.*.

[38] Nigro D., D’Ambrosio V., Sanvitto D., Gerace D. (2022). Integrated quantum polariton interferometry. *Commun. Phys.*.

[39] Scala F., Nigro D., Gerace D. (2024). Deterministic entangling gates with nonlinear quantum photonic interferometers. *Commun. Phys.*.

[40] Muñoz de las Heras A., Tabares C., Schneider J. T., Tagliacozzo L., Porras D., González-Tudela A. (2024). Photonic quantum metrology with variational quantum optical nonlinearities. *Phys. Rev. Res.*.

[41] Haroche S., Raimond J.-M. (2006). *Exploring the Quantum: Atoms, Cavities, and Photons*.

[42] Butcher P. N., Cotter D. (1990). *The Elements of Nonlinear Optics, ser. Cambridge Studies in Modern Optics*.

[43] Cheng R., Zhou Y., Wang S., Shen M., Taher T., Tang H. X. (2023). A 100-pixel photon-number-resolving detector unveiling photon statistics. *Nat. Photonics*.

[44] Eaton M. (2023). Resolution of 100 photons and quantum generation of unbiased random numbers. *Nat. Photonics*.

[45] Mallet F. (2011). Quantum state tomography of an itinerant squeezed microwave field. *Phys. Rev. Lett.*.

[46] Eichler C. (2011). Observation of two-mode squeezing in the microwave frequency domain. *Phys. Rev. Lett.*.

[47] Bergeal N., Schackert F., Frunzio L., Devoret M. H. (2012). Two-mode correlation of microwave quantum noise generated by parametric down-conversion. *Phys. Rev. Lett.*.

[48] ..

[49] Braunstein S. L., Caves C. M. (1994). Statistical distance and the geometry of quantum states. *Phys. Rev. Lett.*.

[50] Paris M. G. A. (2009). Quantum estimation for quantum technology. *Int. J. Quantum Inf.*.

[51] Beckey J. L., Cerezo M., Sone A., Coles P. J. (2022). Variational quantum algorithm for estimating the quantum Fisher information. *Phys. Rev. Res.*.

[52] Fisher R. A., Russell E. J. (1922). On the mathematical foundations of theoretical statistics. *Philos. Trans. R. Soc. London. Series A, Containing Pap. Math. or Phys. Char.*.

[53] Shore B. W., Knight P. L. (1993). The jaynes-cummings model. *J. Mod. Opt.*.

[54] Grynberg G., Aspect A., Fabre C. (2010). *Introduction to Quantum Optics: From the Semi-classical Approach to Quantized Light*.

[55] Kraus B., Büchler H. P., Diehl S., Kantian A., Micheli A., Zoller P. (2008). Preparation of entangled states by quantum Markov processes. *Phys. Rev. A*.

[56] Diehl S., Micheli A., Kantian A., Kraus B., Büchler H. P., Zoller P. (2008). Quantum states and phases in driven open quantum systems with cold atoms. *Nat. Phys.*.

[57] Verstraete F., Wolf M. M., Ignacio Cirac J. (2009). Quantum computation and quantum-state engineering driven by dissipation. *Nat. Phys.*.

[58] Barreiro J. T. (2011). An open-system quantum simulator with trapped ions. *Nature*.

[59] Lin Y. (2013). Dissipative production of a maximally entangled steady state of two quantum bits. *Nature*.

[60] Frisk Kockum A., Miranowicz A., De Liberato S., Savasta S., Nori F. (2019). Ultrastrong coupling between light and matter. *Nat. Rev. Phys.*.

[61] Chang C. W. S. (2020). Observation of three-photon spontaneous parametric down-conversion in a superconducting parametric cavity. *Phys. Rev. X*.

[62] Agustí A., Chang C. W. S., Quijandría F., Johansson G., Wilson C. M., Sabín C. (2020). Tripartite genuine non-Gaussian entanglement in three-mode spontaneous parametric down-conversion. *Phys. Rev. Lett.*.

[63] Casado A. A., Sabín C. (2022). Non-Gaussian entanglement swapping between three-mode spontaneous parametric down-conversion and three qubits. *Phys. Rev. A*.

[64] Qin W., Kockum A. F., Muñoz C. S., Miranowicz A., Nori F. (2024). Quantum amplification and simulation of strong and ultrastrong coupling of light and matter. *Phys. Rep.*.

[65] Devitt S. J., Munro W. J., Nemoto K. (2013). Quantum error correction for beginners. *Rep. Prog. Phys.*.

[66] Cai W., Ma Y., Wang W., Zou C.-L., Sun L. (2021). Bosonic quantum error correction codes in superconducting quantum circuits. *Fundam. Res.*.

[67] Gottesman D., Kitaev A., Preskill J. (2001). Encoding a qubit in an oscillator. *Phys. Rev. A*.

[68] Vasconcelos H. M., Sanz L., Glancy S. (2010). All-optical generation of states for “encoding a qubit in an oscillator. *Opt. Lett.*.

[69] Shi Y., Chamberland C., Cross A. (2019). Fault-tolerant preparation of approximate gkp states. *New J. Phys.*.

[70] Grimsmo A. L., Puri S. (2021). Quantum error correction with the gottesman-kitaev-preskill code. *PRX Quantum*.

[71] Hastrup J., Andersen U. L. (2022). Protocol for generating optical gottesman-kitaev-preskill states with cavity qed. *Phys. Rev. Lett.*.

[72] Gardner J. W. (2024). Stochastic waveform estimation at the fundamental quantum limit. ..

