# Supplementary material for: Improving quantum metrology protocols with programmable photonic circuits
Source: Nanophotonics. 2025 Feb 20;14(11):2075–85. doi: 10.1515/nanoph-2024-0640 (PMC12133255; doi:10.1515/nanoph-2024-0640)
Supplement: Supplementary file 1 — Supplementary Material Details [file j_nanoph-2024-0640_suppl_001.pdf]

Supplementary Material: Improving quantum metrology protocols with programmable photonic circuits

In this Supplementary Material, we provide more details on our manuscript entitled *"Improving quantum metrology protocols with programmable photonic circuits"*. In Sec. SM1 we give more details on how our numerical simulations are carried. In Sec. SM2 we show that our proposal can be experimentally realized in spite of the challenges posed by implementing tunable interactions (Sec. SM2 A) and the restrictions due to coherence times (Sec. SM2 B). In Sec. SM3 we analyze the effect of errors due to imperfect gate control. Sec. SM4 provides a detailed description of the process employed to find the optimal quadrature angles for homodyne detection using the continuous approach. In Sec. SM5 we compare the results obtained for homodyne detection in the programmable approach when one optimizes the quadrature angle or, alternatively, fixes its value. In Sec. SM6 we compute the value of the classical Fisher information (CFI) of the states produced by the programmable approach without including the pre-measurement optimization loop. Finally, in Sec. SM7 we examine the Wigner quasi-probability distribution of the probe states generated with the programmable approach.

SM1. DETAILS ON THE NUMERICAL SIMULATIONS

Here we provide more details on the numerical simulations performed to calculate the results shown in this manuscript.

For both the continuous and the programmable approaches, we perform full state-vector simulations of the photonic quantum states. In practice, one needs to introduce a cutoff for the Hilbert space dimension. In our numerics, we set this cutoff to $2N$ for each mode, where N is the total mean number of photons in the two optical modes. To simulate the phase estimation process, one needs to assume a certain value of the phase φ that one aims at estimating. In our case, we set $\varphi = \pi/3$. To calculate the quantum Fisher information (QFI) according to Eq. (1) of the Main Text, we take $\delta = 10^{-2}$. We have made sure that for such a small value the results of the QFI are converged with respect to δ.

In the programmable approach, we employ the COBYLA method to perform the classical optimization of both the preparation and the pre-measurement parametrized quantum circuits (PQCs). This choice was made after benchmarking COBYLA against different optimization algorithms: BFGS, L-BFGS-B, and SLSQP. COBYLA was the one giving the best results within reasonable computation times. Each optimization starts from random initial parameters close to zero for both the preparation and pre-measurement PQCs (such that, initially, the unitaries of the two PQCs are close to the identity matrix). For each value of the mean photon number N, we initialize both the preparation and pre-measurement PQCs with a single layer. Once the optimization of the two PQCs finishes (after 1000 iterations or a convergence tolerance of 10^{-10}), we add another layer to both PQCs. The new initial parameters for the first layer are the optimal ones, while the initial parameters of the new layer are set to zero. We repeat this process for growing values of d up to $d = 10$. Once this optimization series finishes, we change the initial parameters of the single-layer PQCs and we repeat the process once again from $d = 1$ to $d = 10$. Results are gathered for 60 different sets of initial parameters in the case of the JC ansatz and 40 sets for the Kerr ansatz. The data shown in the Main Text for each value of N and d correspond to the results featuring the largest values of QFI and CFI within the whole pool of optimization runs.

In Fig. SM1 we plot the cost function of the two optimization steps (aimed at maximizing the QFI and the CFI, respectively) of the programmable approach as a function of the iteration of the optimizer, both for the JC and Kerr non-linearity ansätze. The mean-photon number is fixed at $N = 20$. For all instances, we perform a maximum of 1000 iterations, unless convergence (with a tolerance 10^{-10}) is reached before that number. In that case, the optimization is stopped. Although convergence is reached in the majority of cases, it is difficult to assess whether the global minimum has been reached or the optimizer is stuck in a local minimum. To avoid local minima and explore the largest possible amount of the Hilbert space, we employ several instances featuring different initial parameters, as explained in the previous paragraph.

SM2. EXPERIMENTAL REMARKS

A specific challenge posed by the programmable approach consists of the realization of dynamically tunable JC and Kerr non-linearities in the few-photon regime. Besides, a common challenge for deterministic state generation protocols, including the ones presented in this work, consists of achieving sufficiently large coherence times allowing to apply the non-linearities during the required interaction times. In this Section, we show that the two challenges can be overcome in state-of-the-art photonic platforms. Tunable interactions are studied in Sec. SM2 A, while large coherence times are analyzed in Sec. SM2 B.

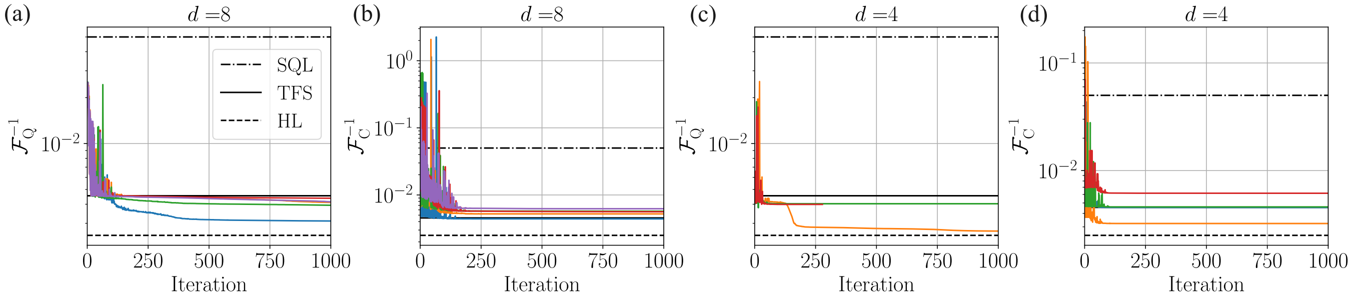

FIG. SM1. Inverse of the QFI $\mathcal{F}_{\mathrm{Q}}^{-1}$ (a,c) and CFI $\mathcal{F}_{\mathrm{C}}^{-1}$ (b,d) as a function of the iteration step of the optimizer, for several instances, each starting from different initial parameters. (a,b) JC non-linearity ansatz for $d = 8$ layers. (c,d) Kerr non-linearity ansatz for $d = 4$ layers.

A. Tunable interactions

Experimental implementations of tunable interactions are already available in certain platforms. On the one hand, in cavity-QED [1–3] it is possible to dynamically tune the coupling strength g of a JC interaction between the cavity mode and a quantum emitter. This can be done by harnessing a so-called Λ transition of the emitter with a Raman laser [4, 5]. In short, in a Λ transition the emitter has three relevant states: two long-lived lower states $|g\rangle$ and $|s\rangle$, as well as one excited state $|e\rangle$. The cavity field couples to the transition $|g\rangle \to |e\rangle$, while the Raman laser couples to the transition $|s\rangle \to |e\rangle$. In this situation, it is possible to engineer an effective coupling between the cavity field and the Raman transition $|g\rangle \to |s\rangle$. Its coupling strength can be tuned by adjusting the detuning and the intensity of the Raman laser.

On the other hand, tunable Kerr non-linearities have been implemented in the microwave regime using superconducting circuits [6–8]. However, it is much more challenging to realize tunable Kerr non-linearities in the few-photon regime at optical frequencies. Recently, Ref. [9] proposed to obtain such a tunability through the coupling of an infrared resonator to intersubband quantum well transition dipoles. However, even if one cannot dynamically tune the nonlinearity strength, an alternative consists of simulating the experiment in a classical computer and then realizing the PQC accordingly, using the optimal parameters found by the optimizer. This removes the requirement of dynamically tunable non-linearities, although one still needs to fabricate components with different non-linearity strengths.

To sum up, for the JC non-linearity an experimental realization of our ideas is within reach in cavity-QED setups. For the Kerr non-linearity, our proposal is already implementable in circuit-QED experiments. In the optical regime, however, an experimental realization is more challenging, but one can rely on first simulating the setup in a classical computer and then building the experiment accordingly.

B. Large coherence times

The coherence time of the specific platform where one wishes to implement our proposal is a limiting factor for the interaction times required to generate the probe states. One should compare the adimensional interaction times shown in Fig. 4 of the Main Text with the ratios g/κ and K/κ between the maximum non-linearity strength achievable (g and K for the JC and Kerr interactions, respectively) and the decoherence rate κ of the photonic mode. Regarding cavity-QED platforms, where the JC non-linearity can be implemented, the largest values of the g/κ ratio are above 10^2 in the microwave regime using superconducting qubits [6, 10–13], while they can reach $g/\kappa \sim 10$ in the optical regime employing atoms and quantum dots in optical cavities [14–19]. On the other hand, state-of-the-art Kerr optical cavities can only reach K/κ of up to 10^{-2} [20, 21], while in the microwave regime it is possible to obtain K/κ of the order of 10^2 [22–24]. Therefore, except for Kerr non-linearities in the optical regime, our proposal should be feasible in state-of-the-art platforms, since the maximum values required are of the order of $\tilde{g} \sim 10$ and $\tilde{K} \sim 1$ (see Fig. 4 of the Main Text).

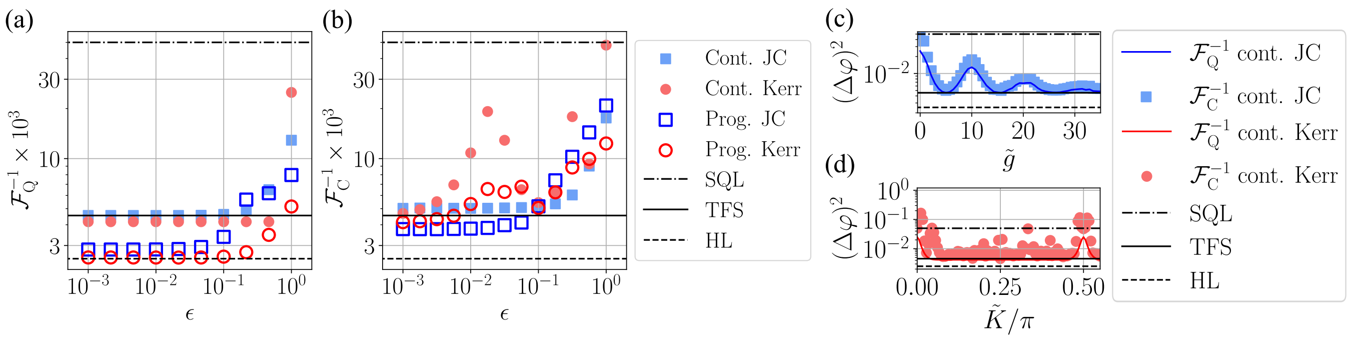

FIG. SM2. (a) Inverse of the QFI $\mathcal{F}_{\rm Q}^{-1}$ for a mean-photon number $N = 20$ as a function of the gate error ϵ for the continuous approach using the JC (solid blue squares) and the Kerr (solid red circles) interactions, as well as for the programmable approach employing the JC (void blue squares) and the Kerr (void red circles) ansätze. In the continuous JC case, the QFI is calculated at its first maximum taking place at $\tilde{g} \simeq 5$. In the continuous Kerr case, the QFI is calculated for the 4-component cat state appearing at $\tilde{K} = \pi/4$. In the programmable cases, we employ the output states generated with $d = 10$ layers in each case. (b) Analog results for the inverse of the CFI $\mathcal{F}_{\rm C}^{-1}$, calculated using homodyne detection. (c) Estimation error $(\Delta\varphi)^2$ obtained with the continuous approach from the inverse of the QFI (blue line) and CFI (blue squares) as a function of the adimensional interaction time $\tilde{g}$ for a mean-photon number $N = 20$. The CFI is calculated for homodyne detection. (d) Analog results for the continuous approach employing a Kerr non-linearity. The red line (red circles) represents the inverse of the QFI (CFI). In the four panels, the black dashed-dotted, solid, and dashed lines correspond to the standard quantum limit (SQL), the results of twin-Fock states (TFS), and the Heisenberg limit (HL), respectively.

SM3. EFFECT OF IMPERFECT GATE CONTROL

This Section considers a realistic implementation of the proposed probe state generation and measurement preparation strategies. In the programmable approach, an important source of errors is the imperfect control when switching the quantum gates on and off. This can hinder the programmable strategy's advantage over continuous time evolution, where one only needs to activate and deactivate the interaction once. In a real experiment, limitations arise from the switching time of the control pulses, such as laser pulses controlling quantum emitters in cavity-QED setups [25], or flux pulses governing tunable transmons in superconducting circuit implementations [26–28]. In general, the switching time $T_{\rm switch}$ needs to be smaller than the characteristic interaction time $T_{\rm int}$ given by the inverse of the energy scale set by the non-linear interactions, i.e., g^{-1} or K^{-1} for the JC and Kerr non-linearities, respectively. We model such a realistic source of gate infidelity by including an error ϵ in the variational parameters of the PQC. Namely, if an errorless variational parameter takes the value θ, we consider $\theta(1+\epsilon)$ as the corrupted parameter. This is done for both the preparation and the pre-measurement PQCs. As a benchmark, we use the continuous approach in which one also introduces the same type of error in the interaction time necessary to generate the probe states. Typical values of ϵ range between $\epsilon \sim 10^{-5} - 10^{-4}$ for Kerr non-linearities in cavity-QED setups [25, 29], as well as for both JC and Kerr non-linearities in superconducting circuits [30], to $\epsilon \sim 10^{-2}$ for JC interactions in cavity-QED [29, 31]. Regarding tunneling and beam splitters, the error varies around $\epsilon \sim 10^{-3}$ and 10^{-2} in the microwave [32] and optical [33] regimes, respectively.

We first analyze the results for the QFI in Fig. SM2(a). Here, we plot the inverse of the QFI $\mathcal{F}_{\rm Q}^{-1}$ for probe states with a mean-photon number $N = 20$ as a function of the error ϵ, for both the continuous and programmable approaches, and for the two interaction types (JC and Kerr). Note that, below $\epsilon \simeq 10^{-1}$, the QFI is barely disturbed by the gate imperfections. This range of values of ϵ coincides with that appearing in state-of-the-art experiments, as discussed in the previous paragraph. We can thus conclude that we typical errors linked to imperfect gate control are not expected to spoil the metrological power of the generated probe states, neither for the programmable nor for the continuous approach. Beyond $\epsilon \gtrsim 10^{-1}$, the metrological power of the generated probe states diminishes. However, such a decrease is larger when one employs the JC non-linearity as the system interaction, both in the continuous and programmable cases. On the other hand, the Kerr nonlinearity is much more noise-resilient. Specifically, in the programmable approach the Kerr non-linearity generates quasi-optimal probe states even for $\epsilon \sim 10^{-1}$. Moreover, in the continuous approach, the values of $\mathcal{F}_{\rm Q}^{-1}$ do not grow with ϵ, and actually they decrease a bit below $\epsilon \sim 1$. This has to do with the dynamics of the QFI as a function of $\tilde{K}$ presented in Fig. 3 of the Main Text. In that Figure, one can see that $\mathcal{F}_{\rm Q}^{-1}$ presents a series of plateaus bordered by peaks. This implies that an error ϵ modifying $\tilde{K}$ within the plateau does not significantly disturb the QFI. Actually, as it happens in Fig. SM2(a), it can decrease $\mathcal{F}_{\rm Q}^{-1}$, as the plateau features some local minima unnoticeable in Fig. 3 of the Main Text. Only when $\tilde{K} + \epsilon$ reaches a value

comparable with the location of the peak, we see an increase in $\mathcal{F}_{\mathrm{Q}}^{-1}$, which is what happens for $\epsilon = 1$. Thus, we can conclude that the continuous strategy employing a Kerr non-linearity is especially error-resilient. Moreover, the advantage of the programmable approach against the continuous one is maintained for imperfect gates with realistic error values.

Regarding the metrological power available in the measurement phase, in Fig. SM2(b) we plot the inverse of the CFI $\mathcal{F}_{\mathrm{C}}^{-1}$ as a function of the error ϵ, for a mean-photon number $N = 20$. We consider a homodyne detection of the $X(\theta)$ generalized quadrature, which is the most experimentally friendly type of measurement analyzed in this work. For the continuous approach, we take the optimal values of the quadrature angle θ obtained in Sec. SM4. For the programmable approach, we choose $\theta = 0$, as the optimizer can effectively rotate the phase space, which was demonstrated in Sec. SM5. Differently from the behavior of the QFI, for the CFI the strategies employing the Kerr non-linearity perform worse than those using the JC interaction, as they deteriorate more rapidly with ϵ. Actually, the JC programmable approach gives a larger metrological advantage than TFS for up to $\epsilon \sim 10^{-1}$, which includes the range of realistic values of ϵ. On the other hand, the $\mathcal{F}_{\mathrm{C}}^{-1}$ provided by approaches based on Kerr non-linearities displays an oscillatory behavior, although growing with ϵ. Such a worse performance of the Kerr non-linearities for homodyne detection can be explained by looking at the $\mathcal{F}_{\mathrm{C}}^{-1}$ of the continuous strategy as a function of the adimensional interaction time. This is shown in Fig. SM2(c,d) for the JC and Kerr interactions, respectively. While the former interaction type results in a smooth dependence of $\mathcal{F}_{\mathrm{C}}^{-1}$ with $\tilde{g}$, very similar to that of the corresponding $\mathcal{F}_{\mathrm{Q}}^{-1}$, Kerr non-linearities lead to fast oscillations of $\mathcal{F}_{\mathrm{C}}^{-1}$ with $\tilde{K}$. This ultimately results in the non-monotonic behavior observed in panel (b).

We conclude that, for the CFI extracted using homodyne detection, the JC programmable approach gives the best results for imperfect gates with realistic values of ϵ. Moreover, the advantage of the programmable strategy over the continuous one is maintained within the realistic range of ϵ.

SM4. OPTIMIZATION OF THE QUADRATURE ANGLES WITH THE CONTINUOUS APPROACH

In this Section, we determine the optimal angles $\theta_{\min}$ for the generalized quadratures $X_i(\theta) = (e^{-i\theta}a_i^\dagger + e^{i\theta}a_i)/\sqrt{2}$ to be measured in homodyne detection using the continuous approach, where $i = 1, 2$ is the index of each photonic mode. This process is carried out independently for each interaction type.

We start by fixing the adimensional interaction time at values producing a minimum of the inverse of the CFI $\mathcal{F}_{\mathrm{C}}^{-1}$. For a mean-photon number $N = 20$, this corresponds to $\tilde{g} \simeq 5$ and $\tilde{K} = \pi/4$ for the Jaynes-Cummings (JC) and Kerr nonlinearities, respectively, see Fig. 2(a) and Fig. 3(a) of the Main Text. For those values of the interaction strength, we then calculate $\mathcal{F}_{\mathrm{C}}^{-1}$ as a function of θ, following the procedure described in Sec. 2 of the Main Text. The results are shown in panels (a,b) of Fig. SM3 for the JC and the Kerr interaction, respectively. In the two cases, $\mathcal{F}_{\mathrm{C}}^{-1}$ displays an oscillatory behavior as a function of θ. For the JC interaction, there are two points located at $\theta_{\min} \simeq 2\pi/3$ and $\theta_{\min} \simeq 5\pi/3$ where $\mathcal{F}_{\mathrm{C}}^{-1}$ takes its global minimum value. Without loss of generality, we use the first one (i.e., $\theta_{\min} \simeq 2\pi/3$) to calculate the optimal generalized quadrature $X(\theta_{\min})$. In the case of the Kerr nonlinearity, there are several values of θ for which $\mathcal{F}_{\mathrm{C}}^{-1}$ reaches its global minimum. Again, without loss of generality, we use the first one, located at $\theta_{\min} = 0.17\pi$, to calculate $X(\theta_{\min})$.

Finally, in Fig. SM3(c) we plot the values of $\theta_{\min}$ where $\mathcal{F}_{\mathrm{C}}^{-1}$ reaches its first global minimum as a function of N. As shown in the figure, their position is independent of N for the two interaction types. Thus, we use the same values of $\theta_{\min}$ to compute the generalized quadratures in homodyne detection regardless of N.

SM5. OPTIMIZATION OF THE QUADRATURE ANGLE WITH THE PROGRAMMABLE APPROACH

In this Section, we study the CFI obtained in homodyne detection using the programmable approach. We compare two strategies: the first one consists on optimizing the angle θ of the generalized quadratures $X_i(\theta) = (e^{-i\theta}a_i^\dagger + e^{i\theta}a_i)/\sqrt{2}$ (where $i = 1, 2$ is the index of each photonic mode) as an additional variational parameter of the pre-measurement PQC. The second strategy only optimizes the pre-measurement PQC, while employing a fixed $\theta = 0$.

The results comparing the two approaches are shown in Fig. SM4, where we plot the inverse of the CFI $\mathcal{F}_{\mathrm{C}}^{-1}$ as a function of the mean-photon number N for the JC and the Kerr ansätze with $d = 5$ layers. For both ansätze, the two optimization strategies produce very similar results. This is because the measurement PQC can effectively rotate the Wigner function of the quantum state in phase space. Thus, it has a similar effect than changing the angle θ of the measured quadrature. This demonstrates that, in the programmable approach, it is not necessary to optimize θ for homodyne detection. This is why in the Main Text we consider results with a fixed $\theta = 0$.

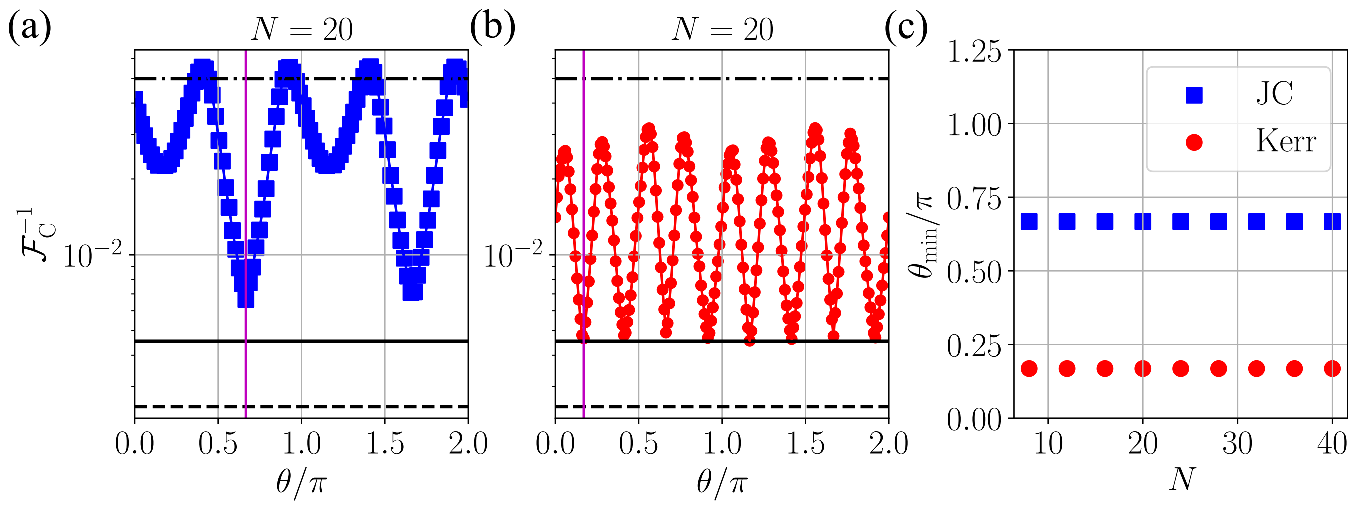

FIG. SM3. (a,b) Inverse CFI $\mathcal{F}_C^{-1}$ as a function of the generalized quadrature angle θ for homodyne detection. The mean-photon number of the initial state is fixed at $N = 20$. The horizontal dashed, solid, and dashed-dotted black lines represent the Heisenberg limit, the twin-Fock states results, and the standard quantum limit, respectively. Squares and circles are the data obtained in the continuous approach using the JC [panel (a)] and the Kerr interaction [panel (b)]. The adimensional interaction time is fixed at $\tilde{g} \simeq 5$ and $\tilde{K} = \pi/4$ for each interaction type. (c) Location $\theta_{\rm min}$ of the minima of $\mathcal{F}_C^{-1}$ signalized by the vertical magenta lines in panels (a) and (b) as a function of N. Blue squares (red circles) are the results for the JC (Kerr) interaction.

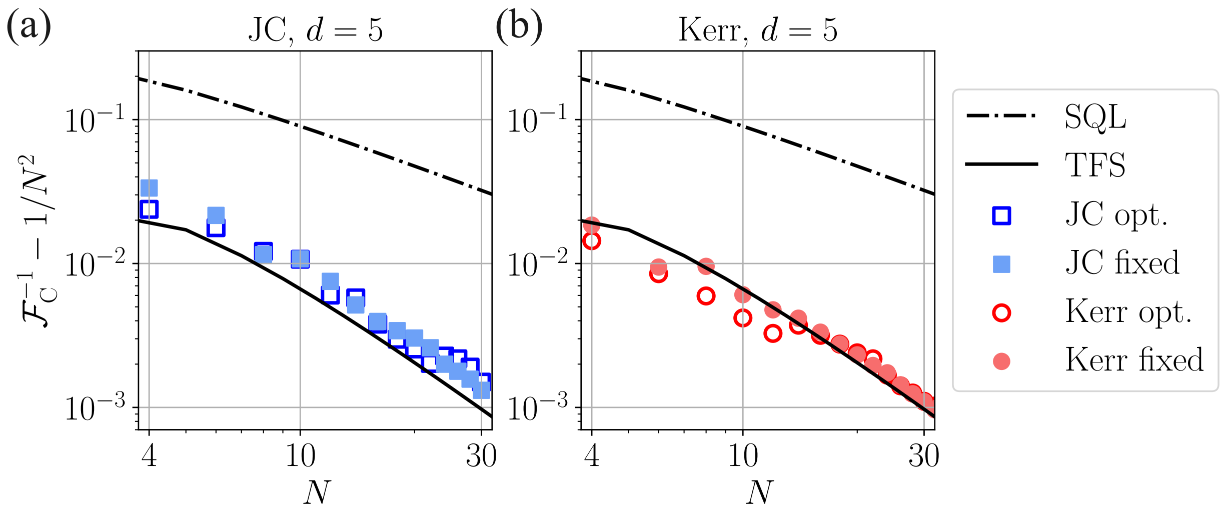

FIG. SM4. Inverse CFI $\mathcal{F}_C^{-1}$ as a function of the mean-photon number N obtained in homodyne detection with the programmable approach using the JC [panel (a)] and the Kerr [panel (b)] ansätze. The number of layers of the preparation and pre-measurement PQCs is fixed to $d = 5$. Solid markers correspond to the results obtained by fixing the quadrature angle to $\theta = 0$, while void markers correspond to the results produced by optimizing the quadrature angle θ as another variational parameter. In the two cases, the pre-measurement PQC is optimized. In both panels, we plot the difference between the data and the Heisenberg limit, $1/N^2$. Solid (dashed-dotted) lines represent the twin-Fock states results (standard quantum limit).

SM6. EFFECT OF THE PRE-MEASUREMENT PARAMETRIZED QUANTUM CIRCUIT

As we explain in Sec. 2 of the Main Text, in the programmable approach we use of two different optimization circuits: The first one is aimed at preparing probe states featuring the largest possible QFI. The second one takes place immediately before the measurement and its objective is to maximize the CFI. Each optimization loop makes use of a different PQC, which we label the *preparation* and the *pre-measurement* PQCs, respectively. However, one may wonder whether it is necessary to perform such a second optimization step, since the probe state generated in

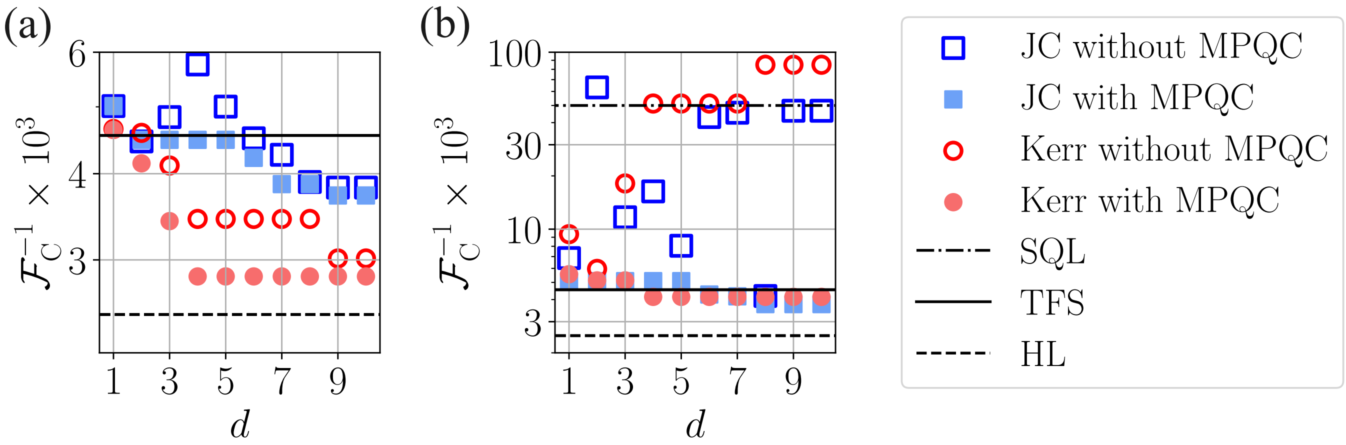

FIG. SM5. Inverse CFI $\mathcal{F}_C^{-1}$ as a function of the number of layers d obtained with the programmable approach using photon counting [panel (a)] and homodyne detection with a quadrature angle $\theta = 0$ [panel (b)]. The mean-photon number is fixed at $N = 20$. Solid markers represent the results obtained by employing the pre-measurement PQC (labelled MPQC), while void markers are computed without the pre-measurement PQC. Blue squares (red circles) correspond to the JC (Kerr) ansatz. Dashed-dotted, solid, and dashed lines represent the standard quantum limit (SQL), the twin-Fock states (TFS) results, and the Heisenberg limit (HL), respectively.

the first optimization can already provide a large value of the CFI.

To assess this question, we examine the effect of the second optimization loop. In Fig. SM5 we plot the inverse of the CFI $\mathcal{F}_C^{-1}$ obtained with and without the pre-measurement PQC. In the first case, the state after the Mach-Zehnder interferometer (MZI, see Sec. 2 of the Main Text for more details) undergoes the second optimization loop using the pre-measurement PQC. This is the method used to compute the results shown in Sec. 5 of the Main Text. However, in the second case, the pre-measurement PQC is absent and the CFI is directly computed using the output state of the MZI. We plot $\mathcal{F}_C^{-1}$ as a function of the number of layers d employed. When the pre-measurement PQC is present, both the preparation and the pre-measurement PQCs have d layers. However, if the pre-measurement PQC is absent, d is the number of layers of the preparation PQC.

Fig. SM5(a) shows the results for photon-counting measurements. For both the JC and the Kerr ansätze, the values of $\mathcal{F}_C^{-1}$ are always larger when the second optimization loop is not performed. This implies a worse metrological performance when the pre-measurement PQC is absent. However, as d increases the two ansätze provide a larger metrological advantage than twin-Fock states (TFS) even in the absence of the second optimization loop. The necessity of the pre-measurement PQC is more evident for homodyne detection, as it is shown in Fig. SM5(b). Here, the quadrature angle is fixed at $\theta = 0$ (see Sec. 2 of the Main Text). When one employs the two optimization loops, both the JC and the Kerr PQCs provide smaller values of $\mathcal{F}_C^{-1}$ than TFS as d increases. However, when the pre-measurement PQC is absent, for both ansätze $\mathcal{F}_C^{-1}$ increases with d, even reaching the standard quantum limit (SQL). This implies that homodyne detection cannot provide any metrological advantage without the second optimization loop, even when one employs the probe states generated with the preparation PQC.

However, it is possible to improve the results of homodyne detection without the pre-measurement PQC by optimizing the generalized quadrature angle θ. In Fig. SM6 we compare the inverse CFI $\mathcal{F}_C^{-1}$ obtained with the pre-measurement PQC and a fixed $\theta = 0$ with the one attained by optimizing the value of θ to maximize the CFI without the pre-measurement PQC. We plot the resulting $\mathcal{F}_C^{-1}$ as a function of the number of layers d of the preparation PQC, which coincides with the number of layers of the pre-measurement PQC when this is included. For the JC ansatz, the values of $\mathcal{F}_C^{-1}$ obtained by performing the optimization loop of the pre-measurement PQC with $\theta = 0$ are still smaller than those obtained by optimizing θ without the pre-measurement PQC. However, especially for $d > 6$, the difference between the values of $\mathcal{F}_C^{-1}$ obtained with the two strategies is much smaller than when the quadrature angle is fixed to $\theta = 0$ and the pre-measurement PQC is not employed (see Fig. SM5). On the other hand, for the Kerr ansatz, the values of $\mathcal{F}_C^{-1}$ obtained by optimizing θ without the pre-measurement PQC are smaller than those obtained by optimizing the pre-measurement PQC with $\theta = 0$. Although the difference between the results of the two strategies is small, optimizing θ without including the pre-measurement PQC can be more efficient in terms of resources since optimizing a single parameter is faster than optimizing $3d$ or $2d$ of them, as required respectively by the JC and Kerr ansätze.

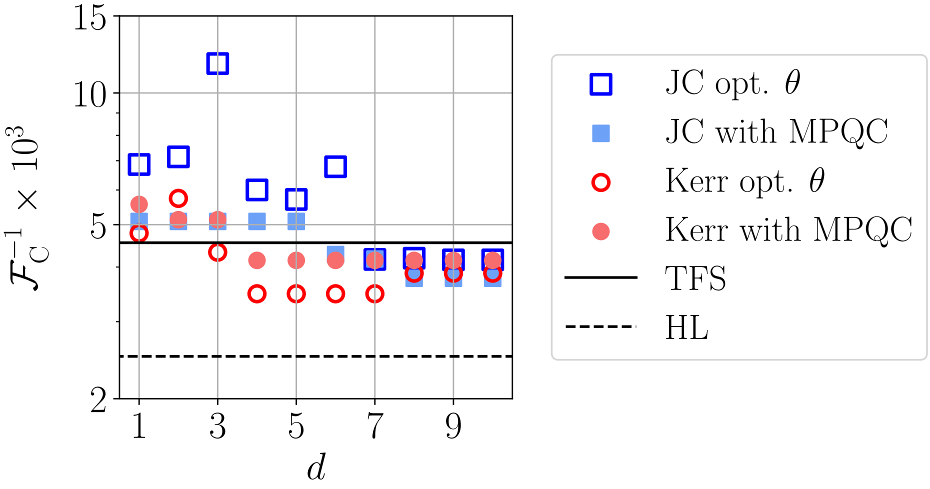

FIG. SM6. Inverse CFI $\mathcal{F}_{\rm C}^{-1}$ as a function of the number of layers d obtained with the programmable approach using homodyne detection. The mean-photon number is fixed at $N = 20$. Solid markers represent the results obtained by employing the pre-measurement PQC (labelled MPQC) and a fixed value of the quadrature angle $\theta = 0$. Void markers are computed by optimizing θ without the pre-measurement PQC. Blue squares (red circles) correspond to the JC (Kerr) ansatz. Solid and dashed lines represent the TFS results and the HL, respectively.

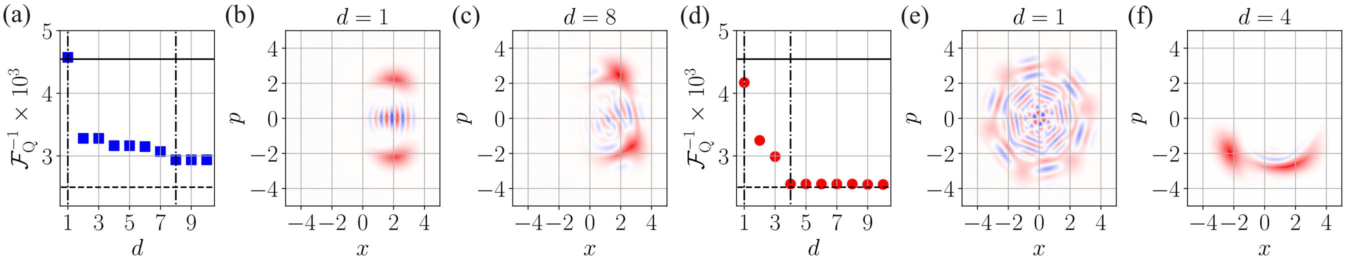

FIG. SM7. Generation of probe states in the programmable approach with a fixed mean-photon number $N = 20$. (a) [(d)] Inverse QFI $\mathcal{F}_{\rm Q}^{-1}$ as a function of the number of layers d. Blue squares (red circles) are the results of the Jaynes-Cummings (Kerr) ansatz. Solid (dashed) lines correspond to TFS (the HL). Dashed-dotted lines signal the values of d for which we plot the Wigner distribution of the output probe states of the parametrized quantum circuit. (b,c) [(e,f)] Wigner distribution in phase space of the states generated by the JC (Kerr) ansatz for $d = 1$ and $d = 8$ ($d = 1$ and $d = 4$).

SM7. WIGNER DISTRIBUTION OF THE PROBE STATES GENERATED WITH THE PROGRAMMABLE APPROACH

In this Section we analyze the probe states generated with the JC and Kerr ansätze in the programmable approach by studying their Wigner quasiprobability distribution. These are displayed in Fig. SM7 for a fixed value of the mean-photon number $N = 20$. For reference, in panel (a) [(d)] we plot the inverse of the QFI $\mathcal{F}_{\rm Q}^{-1}$ obtained using the JC (Kerr) ansatz as a function of the number of layers d of the PQC.

It is interesting to compare the Wigner quasiprobability distribution in phase space of the generated probe states for $d = 1$ and for values of d for which convergence is achieved ($d = 8$ and $d = 4$ for the JC and Kerr ansätze, respectively). As expected, for $d = 1$ the states prepared by each ansatz [see panels (b) and (e)] belong to the same class as the states generated with the continuous approach at values of the interaction strength corresponding to the first minimum of $\mathcal{F}_{\rm Q}^{-1}$. In particular, for the JC ansatz a displaced cat state is produced, while the Kerr ansatz results in a 6-component cat state, both featuring QFI values similar to those of twin-Fock states $|N/2\rangle \otimes |N/2\rangle$.

However, when the number of layers is increased, the output states of the two ansätze, featuring values of $\mathcal{F}_{\rm Q}^{-1}$ close to the Heisenberg limit (HL), become quite different from those produced with $d = 1$. In panels (c) and (f), we plot the Wigner distribution of the states generated by the JC (Kerr) ansatz with $d = 8$ ($d = 4$), when the QFI has converged to its maximal value in each case. Interestingly, these states cannot be associated to any known class of

states, which demonstrates the capability of the optimized PQC to generate non-trivial states.

[1] H. Mabuchi and A. C. Doherty, Science **298**, 1372 (2002), https://www.science.org/doi/pdf/10.1126/science.1078446.
[2] A. Blais, R.-S. Huang, A. Wallraff, S. M. Girvin, and R. J. Schoelkopf, Phys. Rev. A **69**, 062320 (2004).
[3] H. Walther, B. T. H. Varcoe, B.-G. Englert, and T. Becker, Reports on Progress in Physics **69**, 1325 (2006).
[4] D. J. Wineland, C. Monroe, W. M. Itano, D. Leibfried, B. E. King, and D. M. Meekhof, Experimental issues in coherent quantum-state manipulation of trapped atomic ions (1998), arXiv:quant-ph/9710025 [quant-ph].
[5] A. Periwal, E. S. Cooper, P. Kunkel, J. F. Wienand, E. J. Davis, and M. Schleier-Smith, Nature **600**, 630 (2021).
[6] A. Blais, A. L. Grimsmo, S. M. Girvin, and A. Wallraff, Rev. Mod. Phys. **93**, 025005 (2021).
[7] J. J. García Ripoll, *Quantum Information and Quantum Optics with Superconducting Circuits* (Cambridge University Press, 2022).
[8] X. L. He, Y. Lu, D. Q. Bao, H. Xue, W. B. Jiang, Z. Wang, A. F. Roudsari, P. Delsing, J. S. Tsai, and Z. R. Lin, Nature Communications **14**, 6358 (2023).
[9] M. Arias, J. F. Triana, A. Delgado, and F. Herrera, New Journal of Physics **26**, 013003 (2024).
[10] A. Blais, R.-S. Huang, A. Wallraff, S. M. Girvin, and R. J. Schoelkopf, Phys. Rev. A **69**, 062320 (2004).
[11] A. Wallraff, D. I. Schuster, A. Blais, L. Frunzio, R.-. S. Huang, J. Majer, S. Kumar, S. M. Girvin, and R. J. Schoelkopf, Nature **431**, 162 (2004).
[12] R. J. Schoelkopf and S. M. Girvin, Nature **451**, 664 (2008).
[13] X. Gu, A. F. Kockum, A. Miranowicz, Y. xi Liu, and F. Nori, Physics Reports **718-719**, 1 (2017).
[14] K. M. Birnbaum, A. Boca, R. Miller, A. D. Boozer, T. E. Northup, and H. J. Kimble, Nature **436**, 87 (2005).
[15] K. Srinivasan and O. Painter, Nature **450**, 862 (2007).
[16] Y. Arakawa, S. Iwamoto, M. Nomura, A. Tandaechanurat, and Y. Ota, IEEE Journal of Selected Topics in Quantum Electronics **18**, 1818 (2012).
[17] C. Hamsen, K. N. Tolazzi, T. Wilk, and G. Rempe, Phys. Rev. Lett. **118**, 133604 (2017).
[18] H. Takahashi, E. Kassa, C. Christoforou, and M. Keller, Phys. Rev. Lett. **124**, 013602 (2020).
[19] R. M. Kroeze, B. P. Marsh, K.-Y. Lin, J. Keeling, and B. L. Lev, PRX Quantum **4**, 020326 (2023).
[20] A. Delteil, T. Fink, A. Schade, S. Höfling, C. Schneider, and A. İmamoglu, Nat. Mater. **18**, 219 (2019).
[21] G. Muñoz-Matutano, A. Wood, M. Johnsson, X. Vidal, B. Q. Baragiola, A. Reinhard, A. Lemaître, J. Bloch, A. Amo, G. Nogues, B. Besga, M. Richard, and T. Volz, Nat. Mater. **18**, 213 (2019).
[22] J. Koch, T. M. Yu, J. Gambetta, A. A. Houck, D. I. Schuster, J. Majer, A. Blais, M. H. Devoret, S. M. Girvin, and R. J. Schoelkopf, Phys. Rev. A **76**, 042319 (2007).
[23] G. Kirchmair, B. Vlastakis, Z. Leghtas, S. E. Nigg, H. Paik, E. Ginossar, M. Mirrahimi, L. Frunzio, S. M. Girvin, and R. J. Schoelkopf, Nature **495**, 205 (2013).
[24] S. Puri, C. K. Andersen, A. L. Grimsmo, and A. Blais, Nature Comm. **8**, 1 (2017).
[25] H. P. Specht, C. Nölleke, A. Reiserer, M. Uphoff, E. Figueroa, S. Ritter, and G. Rempe, Nature **473**, 190 (2011).
[26] N. Didier, E. A. Sete, M. P. da Silva, and C. Rigetti, Phys. Rev. A **97**, 022330 (2018).
[27] S. A. Caldwell, N. Didier, C. A. Ryan, E. A. Sete, A. Hudson, P. Karalekas, R. Manenti, M. P. da Silva, R. Sinclair, E. Acala, N. Alidoust, J. Angeles, A. Bestwick, M. Block, B. Bloom, A. Bradley, C. Bui, L. Capelluto, R. Chilcott, J. Cordova, G. Crossman, M. Curtis, S. Deshpande, T. E. Bouayadi, D. Girshovich, S. Hong, K. Kuang, M. Lenihan, T. Manning, A. Marchenkov, J. Marshall, R. Maydra, Y. Mohan, W. O'Brien, C. Osborn, J. Otterbach, A. Papageorge, J.-P. Paquette, M. Pelstring, A. Polloreno, G. Prawiroatmodjo, V. Rawat, M. Reagor, R. Renzas, N. Rubin, D. Russell, M. Rust, D. Scarabelli, M. Scheer, M. Selvanayagam, R. Smith, A. Staley, M. Suska, N. Tezak, D. C. Thompson, T.-W. To, M. Vahidpour, N. Vodrahalli, T. Whyland, K. Yadav, W. Zeng, and C. Rigetti, Phys. Rev. Appl. **10**, 034050 (2018).
[28] A. Petrescu, C. Le Calonnec, C. Leroux, A. Di Paolo, P. Mundada, S. Sussman, A. Vrajitoarea, A. A. Houck, and A. Blais, Phys. Rev. Appl. **19**, 044003 (2023).
[29] S. Haroche and J.-M. Raimond, *Exploring the Quantum: Atoms, Cavities, and Photons* (Oxford University Press, 2006).
[30] D. Zhu, T. Jaako, Q. He, and P. Rabl, Phys. Rev. Appl. **16**, 014024 (2021).
[31] M. Uria, P. Solano, and C. Hermann-Avigliano, Phys. Rev. Lett. **125**, 093603 (2020).
[32] Y. Lu, A. Maiti, J. W. O. Garmon, S. Ganjam, Y. Zhang, J. Claes, L. Frunzio, S. M. Girvin, and R. J. Schoelkopf, Nature Communications **14**, 5767 (2023).
[33] J. Mower, N. C. Harris, G. R. Steinbrecher, Y. Lahini, and D. Englund, Phys. Rev. A **92**, 032322 (2015).
